# Surgery

**Published:** 1824-06-01

**Authors:** 


### SURGERY.

1. *Lithotomy.*† Mr. Key, one of the most promising young surgeons of the celebrated school that gave birth to the present lateral ope-

---

† A short Treatise on the Section of the Prostate Gland in Lithotomy; with an Explanation of a safe and easy Method of conducting the Operation ration, has come before the surgical profession, not to propose a novelty, or deviation from, but rather a closer approximation to, the principles of Cheselden's operation, than can be attained by the instruments now commonly employed. A review of Cheselden's principles is, he thinks, required at the present time, when attempts are made by English as well as Continental surgeons, to revive a mode of operating "that presents no advantage under ordinary circumstances—that was discarded by Cheselden—and needs an equal test of time and experience to shew its comparative merit." If want of success in the lateral operation, he observes, has led thus to its disrepute, it becomes a question worth investigating, how far it may be traced to a neglect of those principles which guided the illustrious Cheselden himself.

We have already said that Mr. Key does not come forward with any idea of innovation on the *principle* of the lateral operation, "but merely to suggest an easier *mode* of accomplishing the same object." The success of Cheselden from the year 1731, at St. Thomas's Hospital, where he cut 52 patients in succession, with the loss of only two, attracted the attention of the surgeons of all Europe, who eagerly sought to acquaint themselves with his practice, and closely follow his steps. But his imitators were not so successful as himself, and various improvements, or rather *changes*, were, from time to time, attempted. Among these ranks first, the gorget introduced by Sir Cæsar Hawkins. The fundamental objection to this instrument, in Mr. Key's opinion, is, that from the manner in which it is introduced into the bladder, *it cannot divide the parts according to Cheselden's operation.* Here it becomes necessary to inquire what was the mode in which Cheselden did operate? In his first operation he followed the plan of Frère Jacques, but soon laid it aside. His next operation is thus described by Douglas.

"His knife entered first the muscular part of the urethra, which he divided laterally, from the pendulous part of its bulb to the apex, or first point of the prostate gland, and from thence directed his knife upward and backward all the way to the bladder." P. 4.

This, or second mode, he also abandoned, probably from having wounded the rectum in some of his operations. The following appears to be his third and last mode of operating.

"An assistant holding a long and curved staff, Cheselden, with a pointed convex edged knife, made his usual large external incision through the muscles of the bulb and crus penis, and part of the levator ani, till he could feel with the fore finger of his left hand the prostate gland, at the same time keeping the rectum down, and preventing it being endangered: then, pressing his finger behind the prostate, and feeling the groove of the staff, he turned the edge of his knife upward, pierced the cervix vesicæ, till the edge rested in the groove; and com-

---

on the Principles of Cheselden. Illustrated by Engravings. By C. Aston Key, Surgeon to Guy's Hospital, and to the Magdalen. 4to, pp. 34. Four plates. Longman and Co. May 1824.

pleted the division of the prostate and membranous part of the urethra by withdrawing the knife towards himself." 7.

John Bell describes this last operation of Cheselden's in the following words:—"He struck his knife into the great hollow under the tuber ischii, entered it into the body of the bladder immediately behind the gland, and drawing the knife towards him, cut the whole substance of the gland, and even a part of the urethra;" or, in other words, "cut the same parts the contrary way," alluding to this operation as contrasted with the second. In short, from the concurrent testimonies of those most likely to be acquainted with Cheselden's mode, and which are fairly cited by Mr. Key, there can be no doubt that the prostate gland was divided by that celebrated surgeon in a manner very different from that in which the gorget divides it at the present time. Cheselden's aim was to divide the prostate in the depending part of the left lobe, with a considerable inclination towards the rectum. "The most dexterous operator with the gorget cannot effect this," says Mr. Key;—"the direction which the gorget takes is the very reverse of this—it is directed to be inclined upwards, by which the upper surface of the gland only is sliced off, and the major part of the gland remains whole." 10.

Having made these preliminary sentiments respecting Cheselden's operation, Mr. Key proceeds to adduce his own observations on this important subject.

The form of the staff has always appeared to Mr. Key to present great difficulty in executing the operation on the true principles of the lateral lithotomy.*

"At the part where it serves the purpose of a director it is curved; a form certainly least adapted to convey a cutting instrument with safety where the eye of the operator cannot follow it; and, whether the knife or Gorget be used, difficulties, though of a different kind, present themselves. When the former is propelled along the groove of the curved staff, as in Mr. Martineau's operation, the edge must be turned, if not directly downward, at least not sufficiently towards the left side of the patient to effect the necessary division of the prostate gland; unless the operator be skilful enough to turn the blade and divide the lobe of the gland, in doing which he is obliged to make two incisions, as Mr. Martineau has observed. 'I introduce,' says that gentleman in his valuable paper in the Medico-Chirurgical Transactions, 'the point of my knife into the groove of my staff as low down as I can, and cut the membranous part of the urethra, continuing my knife through the prostate into the bladder; when, instead of enlarging the wound downwards, and thus endangering the rectum, I turn the blade towards the ischium

* "The late Mr. Dease was so impressed with the hazard of passing a cutting instrument along the curve of the staff, that he used to withdraw the staff, after he had opened the urethra, and passing a director through the opening into the bladder, dilated the cervix vesicæ, by introducing the Gorget in the usual manner."

and make a lateral enlargement of the wound in withdrawing my knife. I thus avoid cutting over and over again, which often does mischief, but can give no advantage over the two incisions, which I generally depend upon, unless in very large subjects, when a little further dissection may be required.'

"While quoting this gentleman's description I take the opportunity of mentioning that I had the pleasure of seeing him operate at Norwich in the Summer of 1818, and from his deservedly high character, as a successful Lithotomist, I was induced to pay most minute attention to the several steps of his operation; and I am satisfied from my own observation, as well as from his words, that he conducts his incisions of the several parts precisely on the principles laid down by Cheselden. The depth, extent, and direction of his external incision, and the division of the prostate gland, appear to me to accord in every particular with the operation of the great Lithotomist." 13.

In using the gorget there is the danger of the beak slipping out of the groove of the staff—a danger not imaginary. The operator has to attend to two sensations—the gliding of the beak along the staff—and the resistance made by the prostate gland. While he is overcoming the *latter*, he becomes unconscious of the *former*—and at the time he impales the prostate, loses all certainty of the beak being within the groove. "This difficulty depends as much on the curve of the staff as on the nature of the cutting-gorget, and is one that every candid surgeon must acknowledge frequently to have experienced."

The operator, in raising his hand and giving the first impetus to the gorget, is aware of the hazard he runs, lest the blade slip between the gut and the prostate. By depressing it, he is in danger of thrusting the beak at right angles against the staff, so that the beak cannot run along the groove, or is nearly broken in the attempt. These accidents our author has witnessed, and they are by no means uncommon. Bell and Martineau dwell on the nicety of tact necessary in this part of the operation. "I would recommend," says Martineau, "every young operator to practise the directing of the gorget in the groove of his staff when he holds them in his hand, and he will perceive how easily the beak may slip out, if the convex part of the staff be not familiar to his observation." It is to be recollected that Cheselden never used the staff as a *director*—to the gorget belongs the merit of bringing the staff into this office. "Is it surprising," asks Mr. Key, "that the blind should err in a crooked path?"

In consequence of the inclination upwards of the gorget, which the operator is desired to give, the several sections are made too high—giving rise, according to our author, to the following unavoidable evils:—

"First. The cutting edge of the Gorget is conducted so high under the narrow angle of the pubic arch, as to incur a great risk of wounding the pudic artery; a frequent consequence of the introduction of the Gorget in adults, being, as is well known to surgeons, a profuse gush of arterial blood; and, what is more material, not unfrequently great difficulty in restraining the hæmorrhage after the operation.

"Secondly. In the section of the prostate, the Gorget is carried upward through the large plexus of veins which surround the upper surface of the gland, by which long continued venous hæmorrhage is produced, filling the opening into the bladder with coagula, and preventing the ready exit of urine, both by the wound and penis; thus producing the infiltrations of urine into the cellular membrane, which frequently cause so much irritation after Lithotomy.

"Thirdly. The section of the prostate is made in a direction most unfavourable to the extraction of a calculus. Instead of the free incision made through the depending lobe of the gland by Cheselden, the Gorget merely slices off the upper and narrowest part, leaving the body of the gland, which affords so much resistance to a stone, untouched. This slicing of the gland never affords room enough for a large calculus to pass, and, in the violent efforts to extract it, either the bladder is torn laterally, or, what is worse, the prostate is dragged towards the external wound, and its ligamento-cellular connexion with the arch and ramus of of the pubes destroyed. When the operation is properly performed, that is, when the wound in the prostate is sufficient for the passage of the calculus, the connexion between the prostate and the arch of the pubes remains; and affords an opposing barrier, when the finger is attempted to be thrust upwards by the side of the bladder. The consequences attending the destruction of the attachment of the prostate are worthy of consideration." 18.

To appreciate the consequences of laceration of the prostatic connexions, we must examine accurately the cause of death after lithotomy. This is generally attributed to peritonitis, which Mr. Key avers to be "an extremely rare occurrence, and still more rarely the cause of death," after this operation. During ten years observation at Guy's and St. Thomas's hospitals, he has never seen an unsuccessful case examined after operation, where the cause of death could be fairly charged to the peritoneal inflammation. One pathologic condition, however, he has invariably found to obtain—*suppurative inflammation of the reticular texture surrounding the bladder.* The serious evils to the constitution which result from such a circumstance are illustrated by analogy. Thus in injuries of the scalp, if the wound has penetrated the tendon of the occipito-frontalis, we expect extensive suppuration, not from injury of the tendon, *quoad tendon*, but from laceration or other injury done to the cellular membrane between the tendon and pericranium. So wounds of fasciæ, in the extremities or other parts, are dangerous, not from the injury done to the tendinous fibres, "but from the exquisitely acute inflammatory action set up in the subjacent cellular tissue."

"This reticular membrane may be regarded as an infinite number of serous cavities, communicating with each other, and presenting an incalculable extent of surface. Inflammation spreading rapidly through these cells will quickly affect a surface much greater than that of the peritoneum, and I have witnessed symptoms as acute, pain as severe, and the peculiar depression attending peritonitis as marked in the reticular inflammation, as in the most acute and fatal case of inflammation of the abdominal cavity. The instances I have met with of the texture surrounding the bladder being affected with suppurative inflammation, and terminating fatally, whether arising from Lithotomy or operations for fistulæ in perinæo, are sufficiently numerous to allow me thus to generalize on the subject, and afford a very useful lesson to those who endeavour to profit by examinations after death. In the inspection of those who die after Lithotomy, it is not sufficient to look into the peritoneal cavity, to open the bladder, or to examine the state of the wound; the peritoneum lining the lower part of the abdominal muscles should be stripped off, and the source of evil will then be laid open. The finger will enter a quantity of brick-dust coloured pus in the cellular substance around the bladder, and if considerable force has been used in the extraction of the stone, will readily find its way towards the wound in the perineum; the barrier between the adipose structure of the perineum and the reticular texture of the pelvis being broken down, the suppurative inflammation spreads rapidly along the latter, and may be traced in some cases, between the peritoneum and abdominal muscles, as high as the umbilicus; in one case I have seen it extend to the diaphragm."

21. *Lastly.* Every surgeon fears the slipping of the gorget between the bladder and rectum. Mr. Key much doubts whether this be the direction which the gorget takes when it slips from the staff. In the only instance where he had an opportunity of examining after death, the gorget was found to have slipped from the groove, and was propelled under the arch of the pubes, where it entered the reticular texture above, and to the left side of the bladder. He believes this to be the usual course of the gorget, in such accidents.

In order to obviate the evils attending the gorget and curved staff—and, at the same time, to adhere closely to the operation of Cheselden, Mr. Key uses a straight director, which he finds to answer all the purposes of a common staff, while it is free from its objections. He did not adopt this alteration without first operating at the hospital, both with cutting gorget, and beaked knife. Nor did he lay these aside in consequence of want of success, as the patients recovered—but from the difficulty and hazard attending their introduction—and the general unsuccessful issue of gorget operations compared with Cheselden's method.

Mr. Key's director is straight, with a slight curve near the extremity, to prevent the point being caught in a fold of the bladder while depressing the handle. The groove is made somewhat deeper than in the common staff, to prevent any risk of the knife slipping out. The extremity is not grooved, but rounded like a common sound, to prevent abrasion of the prostate or mucous lining of the bladder. The handle is somewhat larger, to afford a better purchase to the hand. The chief superiority of this instrument consists in its allowing the surgeon to turn the groove in any direction he may wish. Before carrying the knife into the prostate, the groove which has been held downwards for the first incision, "may be turned in an oblique line towards the patient's left side that the operator may think preferable for the division of the prostate." Nor does it preclude the use of the gorget. This instrument may be propelled along the straight groove with more safety than in the curved staff. When the gorget is employed, the corresponding motion of the left hand is not required to carry it into the bladder. The director should be held perfectly quiet while the gorget is propelled along the groove.

The knife resembles, in form, a common scalpel, but is longer in the blade, and is slightly convex in the back near the point, to enable it to run with more facility in the groove of the director. The opening made in the prostate, and also in the perineal muscles, can, in some measure, be regulated by the angle which the knife makes with the director as it enters the bladder. In the majority of cases, our author observes, it will merely be necessary to pass the knife along the director, and having cut the prostate, to withdraw it without carrying it out of the groove, varying the angle according to the age of the patient, the width of the pelvis, and the size of the stone. But we shall give the steps of the operation in Mr. Key's own words.

"An assistant holding the director, with the handle somewhat inclined towards the operator, the external incision of the usual extent is made with the knife, until the groove is opened, and the point of the knife rests fairly in the director, which can be readily ascertained by the sensation communicated; the point being kept steadily against the groove, the operator with his left hand takes the handle of the director, and lowers it till he brings the handle to the elevation described in plate 3, keeping his right hand fixed; then with an easy simultaneous movement of both hands, the groove of the director and the edge of the knife are to be turned obliquely towards the patient's left side; the knife having the proper bearing is now ready for the section of the prostate; at this time the operator should look to the exact line the director takes, in order to carry the knife safely and slowly along the groove; which may now be done without any risk of the point slipping out. The knife may then be either withdrawn along the director, or the parts further dilated, according to the circumstances I have adverted to. Having delivered his knife to the assistant, the operator takes the staff in his right hand, and passing the fore finger of his left along the director through the opening in the prostate, withdraws the director, and exchanging it for the forceps, passes the latter upon his finger into the cavity of the bladder.

"In extracting the calculus, should the aperture in the prostate prove too small, and a great degree of violence be required to make it pass through the opening, it is advisable always to dilate with the knife, rather than expose the patient to the inevitable danger consequent upon laceration." 30.

In the case, in which Mr. Key lately operated in this manner, the stone was very readily extracted, though of a large size, from the bladder of a boy four or five years old, in the presence of Messrs. Travers, Green, and Tyrrell.

We sincerely hope Mr. Key's modification may supersede the dark and dismal operation by the gorget. Four excellently engraved plates are appended to the work, which, we trust, will not be the last which we shall receive from the same pen.

---

2. *Amputation of the Lower Jaw.* This terrible operation was lately performed by Professor Lallemand, of Montpellier, under circumstances at once unexpected and appalling. The operator evinced, on this trying occasion, a firmness of mind, a quickness of apprehension, and a fertility of resource, which are no trifling advantages in the hour of difficulty and danger.

The patient was afflicted with a disease—a cauliflower looking mass or tumour, involving the lower jaw and neighbouring parts, that left no other resource but a bold and bloody operation, which was performed by M. Lallemand, at the Saint Eloi Hospital, on the 11th October, 1823. The steps of the operation we shall not detail. Suffice it to say, that the tumour and maxilla inferior were removed by knife, saw, and chissel, amidst a deluge of blood. But this was not the worst. Towards the close of the operation, and before any of the numerous vessels could be secured, the man, after making some laborious efforts to breathe, fell down apparently dead on the floor. "*Ces apparences de mort, l'aspect hideux et effrayant de cette figure mutilée et cadavareuse jeterent l'effroi et la consternation parmi les eleves. Deux ou trois, seulement, furent en etat de m'aider jusqu'au bout.*" Seeing the blood still projecting from the mouths of twenty vessels, M. Lallemand properly concluded that this was not death, nor even syncope from loss of blood. He instantly suspected that the air passage was blocked up with coagula, and on putting his finger into the pharynx, found the tongue reversed into the back part of the mouth, (renversée dans l'arriere-bouche,) and the posterior face of the larynx squeezed against the cervical vertebræ. He quickly perceived that the section of the genio-glossi, genio-hyoidei, and mylo-hyoidei muscles had allowed their antagonists to convulsively produce this "bouleversement" in the situation of the tongue and larynx, and prevent the ingress of air into the lungs, causing, in fact, suffocation. He thus found himself between the two horns of a dilemma—asphyxia on one hand, and hæmorrhage on the other. He rapidly concluded, in his own mind, (and rightly concluded,) that he could not replace the blood if it once issued from the vessels, (je pensais que je ne pourrais pas faire rentrer le sang dans les vaisseaux une fois qu'il serait sorti) although he might be able to introduce air into the lungs a considerable time after they had ceased to perform their functions. He therefore disregarded the suffocation, and attended only to the hæmorrhage. The white-hot cautery was successively applied to the bleeding vessels, and although each application caused the blood to spring with redoubled force for a moment, the hæmorrhage soon ceased. During these successive cauterizations the patient exhibited scarcely any signs of life, and the few students who were able to bear the sight, were astonished at the sang-froid with which our author persevered in searing the wounds of a man whom they considered as dead. They could not, of course, have been aware of the train of thoughts that passed through the operator's mind previously. The hæmorrhage stopped, our author seized a bistoury, and plunged it into the air-passage, between the thyroid and cricoid cartilages. Instantly the air rushed into the lungs—the patient breathed—opened his eyes and looked about, as if awaking from a profound sleep—and immediately raised himself on his breech. The wound was dressed, and a canula left in the tracheal orifice. No bad accident occurred, and the man perfectly recovered. He could make himself understood, notwithstanding the loss of his jaw—and by pushing the food, finely cut, up into the pharynx with his fingers, he managed to take plenty of nourishment, so that he left the hospital in a state of embonpoint.—*Journal Complement. Mars.*

We think the detail of the above case will justify the eulogies which we passed on M. Lallemand's firmness and fortitude in this embarrassing scene.

---

3. *Priapism.* A distressing case of this kind occurred in the practice of Mr. Callaway, and is detailed in the April number of the Med. Repository. It lasted sixteen days, in spite of every medicine and means that could be suggested. At length a lancet was pushed into the left crus of the penis, below the scrotum, and a large quantity of dark grumous blood, with numerous small coagula, escaped. For several days the aperture continued to discharge blood mixed with pus into the poultices; in a few days after which, the man was able to return to his work. Impotency was the consequence of this disease.

---

4. *Paracentesis Capitis.* Mr. Brown, of Preston, has related a case of this kind in No. 300 of the Medical and Physical Journal. The patient was a female child, five years of age, who had been ill for some weeks before Mr. Brown saw her. When visited on the 16th September, 1823, the child was perfectly quiet, eyelids half-closed, pupils insensible to light, head very large, fontanels enlarged, and the integuments much distended, with a sense of fluctuation on pressure. The next day an operation was proposed to the mother, and consent, with great difficulty, obtained. An incision with a lancet was made through the integuments at the frontal angle of the right parietal bone, and then a trochar introduced. Nine ounces of a clear lemon-coloured fluid issued forth. A bandage was then applied pretty tight round the head. The child was rather more alive to external impressions after the operation. Blue ointment was ordered to be rubbed in, and calomel and digitalis to be given internally. 18th. Pulse 170—bowels open—child lively. 19th. Pulse lower, and child still more lively. 20th. Had convulsions last night—hemiplegia (in the mother's opinion) this morning, on the right side. 21st. The child playful—motions of the limbs all free—eyes susceptible to light—pulse 165—bowels open—fæces of a yellow colour. 24th. Strabismus—vertex prominent—fluctuation. Operation proposed, but not consented to. We need not detail the daily reports till the 16th of October, when it is stated that the child lies comatose, being "blind with its eyes open," to use the expression of its mother. The integuments of the cranium are exceedingly tense. The operation again performed, but nothing except blood escaped through the canula. Another puncture was then made about an inch from the former, when blood and pieces of brain came away, and then better than nine ounces of sero-sanguineous fluid. After the operation the child got sick and vomited; but subsequently became rather lively. 18th. The child is completely lethargic and paralytic. The canula was again introduced, and six ounces of fluid drawn off. This operation was followed by a temporary revival. 20th. The child again comatose. The operation performed, and four ounces of fluid drawn off. On the 22d a spontaneous evacuation took place, after which, "the child recovered most surprizingly." On the 24th the child died.

*Dissection.* Cerebrum very pale and soft—great quantity of water in the ventricles—choroid plexus very pale, and containing a cluster of hydatids. "The quantity of fluid removed by the examination amounted to about a quart."

We must confess that a careful perusal of this case, and an unbiassed reflexion on the pathology of the disease, do not bring us to Mr. Brown's conclusion, that—"paracentesis capitis, in the chronic form of hydrocephalus, holds out *great hopes* of eventual success, provided the operation be early employed." Do we see anything like success attending the removal of water from the chest, or even the abdomen, by an operation? And is not success much more likely to follow paracentesis in these last cases than where water is collected in the ventricles or under the membranes of the brain? By this observation we are far from wishing to detract from the boldness and zeal of Mr. Brown.

---

5. *Obliterated Urethra.* Mr. Maclure (of Glasgow) relates the case of a man, aged 40, who, many years previously, had suffered severely from mismanaged or obstinate venereal ulcers. When he applied to our author, the lower segment of the glans penis was found wanting—there was a fistulous opening in the perineum, through which the urine had flowed for years, and from which a probe could be passed backwards towards the bladder, but not forwards towards the glans, on account of an obliterated portion of the canal, about an inch and a half in extent. Down to this obliteration a bougie could be introduced from above. The obliteration occupied, in our author's estimation, the place of the bulb, and perhaps a small portion of the membranous part of the urethra. There was a grave debate between Mr. Maclure and his partner, whether an attempt to remedy the defect should be made, or the man left *in statu quo.* At length an argument was started, which instantly decided their determination. It was suggested that, if *they* did not attempt the cure, some others might:—and be successful. This argument was conclusive in favour of an operation. The next point of discussion respected the means. The caustic bougie of Home was carried against the plans recommended by Dessault, Physic, Bell, and others. The armed bougie indeed seems to be in higher estimation with Mr. Maclure than with the surgeons on this side of the Tweed. The operations were commenced on the 15th of November, 1822, and practised every second day till the 16th of January following, when the bougie "evidently passed onwards to the bladder." On the following day a catheter was passed through the new channel into the bladder, and urine was discharged by it for the first time during a period of sixteen years. For some time things went on well; but abscesses formed, and great impediments were thrown in the way of anticipated success. At length, by the middle of June, the man was able to make water *per vias naturales,* and perform other important functions, the fistulous opening being firmly pressed upon with the finger.

When our author was projecting an ingenious mode of closing the fistulous opening by means of "an instrument constructed upon the principle of a cheese *pale,* as it is called, or of a wright's *wimble,* which being introduced into the fistula and turned round, might so denude its sides of skin, as to make their adhering to one another a matter of certainty," an event occurred which dissolved his benevolent schemes into air—thin air. "Two days ago my patient found it necessary to leave town, under circumstances which exclude all hope of his return to his wife and friends; but which prove, in the most conclusive way, the completeness of the cure, and his perfect confidence in his restored virility." *Ed. Journal,* No. 2.

Thus, then, the removal of a *physical* defect only led to the commission of a *moral* crime! And two months' boring of the urethra by a *caustic bougie* only *opened the way* to adultery and the desertion of an affectionate wife! Oh! Sir Everard! How much have you to answer for in the land of spirits!

---

5. *Extirpation of the Parotid Gland.* In the *Archives Generales* for January, M. Berard has reported the above operation as performed by M. Beclard in LA PITIE on the 19th of August, 1823. The patient was a paper-stainer, who entered the hospital for a cancerous ulceration of the parotid gland. The disease had commenced eight years previously; but, though indolent for a long time at first, it had laterly increased rapidly and become the seat of lancinating pains. It was fixed, and when he entered the hospital, it was of considerable elevation. It raised the lobe of the ear above, apparently involving the cartilaginous portion of the auditory canal—downwards it extended more than an inch below the angle of the jaw—backwards it adhered to the sterno-mastoid muscle—and anteriorly it covered a great part of the masseter. It was ulcerated in two places.

*Operation.* The tumour was inclosed by two curved incisions, one inferior and one posterior. That part situated over the masseter was easily dissected off. Then an attempt was made from below upwards; but a projection of its substance plunged deeply behind and beneath the internal pterigoid, the removal of which would, the operator thought, endanger hæmorrhage. M. B. therefore decided to dissect upwards, by striking the bistoury into the structure of the tumour itself, on a level with the projection, while the instrument divided the cellular tissue connecting it with the adjoining parts. Half the inferior circumference of the cartilage contributing to form the auditory canal was removed by the first dissection. Numerous arteries were tied at this stage of the operation, and M. Beclard continued the extirpation of the remainder of the tumour. When nearly the whole scirrhous mass was removed by successive slices, a large jet of arterial blood announced the section of the external carotid, or one of its large branches. A finger was put on the place whence the jet issued, and the vessel was seized with the forceps, while a needle with a double ligature was passed around it. An assistant tied the vessel above and below the wound in it, which was lateral. The artery was then held forward out of the way, whilst the surgeon completed the extirpation of great part of the tumour. One small projection of the tumour placed before the cervical vertebra was left, on account of its proximity to the internal jugular vein. M. Beclard passed two ligatures beneath this part, by means of a needle, tying the one at the superior the other at the inferior extremity. The wound, which formed a tremendous chasm, was dressed forthwith. Nothing particular occurred for the first days after the operation. All that side of the face was bereaved of expression. The right eye remained open, and, in consequence of being dry, became inflamed. The suppuration was going on kindly, and healthy granulations covering the wound when, on the 12th day, the patient experienced rigors, followed by fever. Erysipelatous inflammation attacked the neighbouring parts, and delirium supervened. When this subsided, taciturnity ensued, and ultimately mental alienation, ending in death, better than three months after the operation, the wound being closed except in one place near the ear, where it was again assuming the cancerous appearance.

On dissection, the pia mater was found injected, water in the ventricles, some pus in the meatus auditorius. The external carotid artery was found to terminate in cellular membrane resulting from cicatrization of the wound—the internal jugular vein was obliterated at the same place.

---

7. *Tetanus Traumaticus.* Mr. Liston lately amputated the lacerated hand of a boy in whom tetanus took place some days after the laceration. The branch of the median nerve going to supply the thumb was found torn two thirds across, and its extremity inflamed and thickened for nearly an inch. The opisthotonos went off after the operation, and the jaw could easily be opened, so that sanguine expectations of recovery were entertained. But the boy was obstinate, and would not take his medicines (calomel and opium with purgatives) and the spasms returned. He died three weeks after the accident, and eight days after the amputation. Mr. Liston thinks that had the amputation been earlier performed, before the disease was completely established, success would have been more probable.—*Ed. Journal.*

---

8. *Denuded Nerves of the Teeth.* Mr. Koecker, an eminent dentist from the United States, and who is now settled in this metropolis, has published a paper in our respected cotemporary (*the Med. and Phys. Journal for March*) on the "treatment of the nerves and lining membrane of the teeth, when denuded," from which we shall make an extract or two.

Mr. K. considers the remedies which have been recommended for these diseases as "cruel and contrary to reason." These remedies are, the knife, acids, and actual cautery, with the view of destroying the nerve of the tooth and its investing membrane. The pain of this operation "is so intense and protracted," that few patients are willing to submit to it; but this is not all—in some irritable constitutions the operation is followed by "an inflammation of the whole mouth, which becomes soon concentrated upon the parts near the affected tooth, where tumefaction and suppuration take place." This requires the extraction of the tooth. But suppose these accidents do not take place, "a tooth which has been deprived of its vitality by the destruction of its nerve, acts upon the parts with which it is in immediate contact as a foreign dead body. It produces all the evil effects which are usually the consequence of a dead root of a tooth, but in an infinitely greater degree.

"In treating a case of the kind under consideration, I have always held it a principal object to preserve the life of the lining membrane, and thus to save the life of the whole tooth.

"My indications for the attainment of this purpose are—

"1. To put a stop to the caries, and consequently to prevent the irritation upon the internal membrane of the tooth.

"2. To suppress the hæmorrhage, and cure the wound of the membrane, if it be wounded.

"3. To protect the membrane artificially against the action of all foreign or external agents.

"To obtain the first of these objects, I cut away all the unsound or dead parts of the tooth, so that every part of the carious cavity be sound, firm, and white. I give the cavity the best possible form for the reception of the metal and its firm retention. I next wash it out with a little lock of cotton, fastened to a straight elastic probe, dipped in warm water. The cavity must be very carefully freed from the small pieces of bone that may stick to it.

"If the lining membrane is not wounded, I immediately plug the cavity with metal; but, if there exist a wound and hæmorrhage, I resort to the treatment for the second indication, by which I endeavour to put an immediate stop to the bleeding, and to cure the wound. For this purpose, I was for some time, in the commencement of my practice, in the habit of employing mild acids and styptics; but I did not find those applications answer any good purpose. The first act destructively on the surrounding parts, and the second were not sufficiently certain in their operation. I therefore soon abandoned such remedies, and resorted to the actual cautery. By this application I readily effect an artificial cicatrization of the wound and a stoppage of the hæmorrhage.

"I require for the operation the following apparatus—1. A thin iron wire, fastened to an ivory handle. The extremity of this wire I file to about the tickness of the exposed surface of the nerve; and bend the wire in such a manner as to enable me to touch the exposed part of the membrane, without coming in contact with any other part of the mouth. 2. A tallow candle, with a thick wick. I direct the patient to discharge all the saliva he may have in his mouth; and then let him incline his head backwards against the head-support of my operation chair. I put the candle into his left hand, and direct him to hold it so that the flame of it may be in a position horizontal with his mouth, and about eight inches from it; I now place myself on the right side of the patient, and, holding his lips with my left hand, so that the instrument may not touch them, I again dry the cavity as perfectly as possible with a lock of cotton fastened to the point of the cauterising wire. Having effected this, I throw away the cotton from the extremity of the wire, and make it red-hot in the flame of the candle. With the wire thus heated I touch the denuded part very rapidly, so that its surface forms an eschar; without, however, suffering it to penetrate deeply into the substance of the bone or the cavity, for this would inevitably bring on suppuration and destruction of the nerve. The nerve must be touched very quickly, and the cautery be perfectly *red* hot. It is sometimes necessary to apply it two or three times before the parts are sufficiently cauterised. When the cautery is red hot it acts suddenly, and almost entirely without pain: but, when it is merely hot, much pain and inflammation is generally produced." 193.

When the hæmorrhage is arrested, Mr. K. leaves the further healing to nature. The last step is to secure the nerve against the influence of the air, by filling up the cavity of the tooth with metal.

"The nerve, which before cauterisation, assumes a fleshy appearance, looks, after this operation, like a black point. I take care not to disturb this point; for, if the black scar be removed, a new wound will be formed, and bleeding induced. I now take a small plate of very thin lead leaf, and lay it upon the denuded nerve and the immediately surrounding bony parts. I next fill up the whole cavity very carefully with gold. In order that success may attend this operation, it is absolutely necessary to make the proper curative applications with the utmost degree of exactness and care, since the smallest error in this will inevitably bring on a destruction of the life of the tooth, and consequently its loss. Thus, for instance, the whole of the operation will prove abortive, if the smallest particle of dead matter, or inflamed bony substance, is suffered to remain in the cavity. Such foreign dead matters, left in contact with the living tooth, soon acquire corrosive qualities, and act destructively upon the contiguous parts, by irritating and inflaming them. If any particles be allowed to remain in contact with the nerve, it is impossible that the operation can succeed properly. Even the smallest quantity of blood left in the cavity soon becomes corrosive, and prevents the success of the operation. All kinds of moisture must be removed before introducing the metals, as the two contiguous metals might produce galvanic effects, if there be any intervening moisture, and thus create a source of irritation and inflammation to the nerve.

"When, therefore, the cavity is once completely cleared of the loose particles of matter, and made perfectly dry, the metal should be quickly introduced, without giving it time to become moist again from the natural exhalations in the mouth. The gold should, of course, be pressed as firmly and compactly into the cavity as possible, in order to prevent the insinuation of any moisture under it. It is here that the skill of the operator becomes of the highest importance; for, if he has been successful in preserving the life of the nerve, the duration of the tooth depends on the skilful manner in which it is plugged; and this is one of those operations by which one dentist may have an opportunity of displaying his superiority over another." 194.

We wish every success to Mr. Koecker; and can say that he has brought with him testimonials from America, of the most honorable kind.

---

9. *Intestinal Tympanites.* Dr. Levrat, Physician of the Hotel Dieu of Lyons, has related a curious case of this kind which was cured by puncturing the intestine, and setting free its gazeous contents. The patient was Madam Lepin, to whom he was called on the 25th day after a painful delivery, in which the fourchette was torn. He found the patient apparently dying; with facies hippocratica, small, creeping pulse, cold extremities, and tumefied abdomen, which was very painful on pressure. Cordials, anodynes, and emollients were first prescribed, and removed the most urgent symptoms; but intestinal tympanites remained, and continued, in spite of various medicines, to increase to an enormous extent. The convolutions of the bowels could be distinctly traced, and the air could be heard rushing from one knuckle of intestine to another. Emaciation had made great progress. Dr. L. determined on an operation. A very delicate trochar was constructed, not much larger than a darning needle, and about two inches in length. This was pushed through the parietes of the abdomen, on the right side, midway between the navel and the spine of the ilium, and into a projecting roll of inflated intestine. Immediately the stilet was removed, a quantity of gas rushed out through the canula, and the belly experienced a great reduction in size. The patient passed a good night, and next day a large quantity of hardened scybala came away, with infinite relief to the lady. From this time she went on well, and rapidly recovered. We forgot to mention that the canula (corked) was left in for some hours, and then some more gas drawn off.

This operation has been practised before, for tympanites, but not, we believe with success—probably because the wound in the parietes of the belly and in the intestine was made with too large an instrument, in consequence of which gas or other matters may have escaped from the intestinal tube into the general cavity of the peritoneum, and there excited inflammation. In Dr. Levrat's operation, the instrument was nearly as fine as that used in acupuncture, and consequently the wound in the intestine was so small as not to permit the escape of any intestinal contents into the cavity of the peritoneum, when the trochar was withdrawn.—*Bulletin des Sciences.*

